# Supplementary material for: Maternal obesity may disrupt offspring metabolism by inducing oocyte genome hyper-methylation via increased DNMTs
Source: eLife. 2024 Dec 6;13:RP97507. doi: 10.7554/eLife.97507 (PMC11623932; doi:10.7554/eLife.97507)
Supplement: Supplementary file 1. [file elife-97507-supp1.docx]

**Table S1 Some information of whole genome bisulfite sequencing.**

| Samples | Total reads | Mapped reads | Unique Mapping rate (%) | Mean Sequencing Depth | Conversion Rate (%) |
| --- | --- | --- | --- | --- | --- |
| CD1 | 102971225 | 33856938 | 32.88 | 2.46 | 99.449 |
| CD2 | 99413634 | 37359643 | 37.58 | 2.69 | 99.458 |
| HFD1 | 87762792 | 32867165 | 37.45 | 2.48 | 99.477 |
| HFD2 | 85697050 | 31613641 | 36.89 | 2.45 | 99.489 |
| HFD3 | 104081620 | 35991424 | 34.58 | 2.75 | 99.501 |
