# Supplementary material for: Maternal obesity may disrupt offspring metabolism by inducing oocyte genome hyper-methylation via increased DNMTs
Source: eLife. 2024 Dec 6;13:RP97507. doi: 10.7554/eLife.97507 (PMC11623932; doi:10.7554/eLife.97507)
Supplement: Supplementary file 2. [file elife-97507-supp2.docx]

**Table S2 KEGG pathway analysis of metabolism-relative genes**

| **KEGG class** | **Pathway** | **Out (147)** | **All (9198)** | **Pathway ID** | **Genes** |
| --- | --- | --- | --- | --- | --- |
| Amino acid metabolism | Glycine, serine and threonine metabolism | 2 | 42 | ko00260 | ENSMUSG00000017713;ENSMUSG00000024039 |
| Amino acid metabolism | Tyrosine metabolism | 1 | 40 | ko00350 | ENSMUSG00000055301 |
| Amino acid metabolism | Cysteine and methionine metabolism | 1 | 54 | ko00270 | ENSMUSG00000024039 |
| Amino acid metabolism | Valine, leucine and isoleucine degradation | 1 | 57 | ko00280 | ENSMUSG00000062908 |
| Amino acid metabolism | Lysine degradation | 1 | 66 | ko00310 | ENSMUSG00000002028 |
| Carbohydrate metabolism | Inositol phosphate metabolism | 4 | 73 | ko00562 | ENSMUSG00000039936;ENSMUSG00000023805;ENSMUSG00000028894;ENSMUSG00000025477 |
| Carbohydrate metabolism | Amino sugar and nucleotide sugar metabolism | 2 | 51 | ko00520 | ENSMUSG00000031387;ENSMUSG00000028671 |
| Carbohydrate metabolism | Citrate cycle (TCA cycle) | 1 | 32 | ko00020 | ENSMUSG00000061838 |
| Carbohydrate metabolism | Pentose phosphate pathway | 1 | 33 | ko00030 | ENSMUSG00000021957 |
| Carbohydrate metabolism | Galactose metabolism | 1 | 34 | ko00052 | ENSMUSG00000028671 |
| Carbohydrate metabolism | Propanoate metabolism | 1 | 34 | ko00640 | ENSMUSG00000061838 |
| Carbohydrate metabolism | Glycolysis / Gluconeogenesis | 1 | 70 | ko00010 | ENSMUSG00000055301 |
| Energy metabolism | Nitrogen metabolism | 1 | 17 | ko00910 | ENSMUSG00000028463 |
| Energy metabolism | Oxidative phosphorylation | 1 | 125 | ko00190 | ENSMUSG00000022450 |
| Global and overview maps | Fatty acid metabolism | 3 | 61 | ko01212 | ENSMUSG00000062908;ENSMUSG00000007783;ENSMUSG00000025153 |
| Global and overview maps | Biosynthesis of amino acids | 3 | 81 | ko01230 | ENSMUSG00000021957;ENSMUSG00000017713;ENSMUSG00000024039 |
| Global and overview maps | Carbon metabolism | 2 | 124 | ko01200 | ENSMUSG00000021957;ENSMUSG00000061838 |
| Glycan biosynthesis and metabolism | Various types of N-glycan biosynthesis | 2 | 40 | ko00513 | ENSMUSG00000043998;ENSMUSG00000020346 |
| Glycan biosynthesis and metabolism | N-Glycan biosynthesis | 2 | 51 | ko00510 | ENSMUSG00000043998;ENSMUSG00000020346 |
| Glycan biosynthesis and metabolism | Other glycan degradation | 1 | 18 | ko00511 | ENSMUSG00000033857 |
| Glycan biosynthesis and metabolism | Glycosylphosphatidylinositol(GPI)-anchor biosynthesis | 1 | 25 | ko00563 | ENSMUSG00000014245 |
| Glycan biosynthesis and metabolism | Glycosphingolipid biosynthesis - lacto and neolacto series | 1 | 28 | ko00601 | ENSMUSG00000021360 |
| Lipid metabolism | Ether lipid metabolism | 4 | 48 | ko00565 | ENSMUSG00000030703;ENSMUSG00000023913;ENSMUSG00000027695;ENSMUSG00000049721 |
| Lipid metabolism | Sphingolipid metabolism | 3 | 47 | ko00600 | ENSMUSG00000021263;ENSMUSG00000057342;ENSMUSG00000049721 |
| Lipid metabolism | Fatty acid degradation | 3 | 52 | ko00071 | ENSMUSG00000062908;ENSMUSG00000055301;ENSMUSG00000007783 |
| Lipid metabolism | Fatty acid biosynthesis | 1 | 18 | ko00061 | ENSMUSG00000025153 |
| Lipid metabolism | Steroid biosynthesis | 1 | 22 | ko00100 | ENSMUSG00000024799 |
| Lipid metabolism | Glycerophospholipid metabolism | 2 | 101 | ko00564 | ENSMUSG00000025357;ENSMUSG00000027695 |
| Lipid metabolism | Glycerolipid metabolism | 1 | 65 | ko00561 | ENSMUSG00000025357 |
| Lipid metabolism | Steroid hormone biosynthesis | 1 | 103 | ko00140 | ENSMUSG00000024365 |
| Metabolism of cofactors and vitamins | Thiamine metabolism | 1 | 16 | ko00730 | ENSMUSG00000026807 |
| Metabolism of cofactors and vitamins | Porphyrin and chlorophyll metabolism | 1 | 43 | ko00860 | ENSMUSG00000028684 |
| Metabolism of cofactors and vitamins | Nicotinate and nicotinamide metabolism | 1 | 44 | ko00760 | ENSMUSG00000029063 |
| Metabolism of cofactors and vitamins | Retinol metabolism | 1 | 102 | ko00830 | ENSMUSG00000055301 |
| Metabolism of other amino acids | Glutathione metabolism | 1 | 68 | ko00480 | ENSMUSG00000022562 |
| Nucleotide metabolism | Purine metabolism | 4 | 139 | ko00230 | ENSMUSG00000021699;ENSMUSG00000026807;ENSMUSG00000021684;ENSMUSG00000042638 |
| Xenobiotics biodegradation and metabolism | Drug metabolism - cytochrome P450 | 1 | 74 | ko00982 | ENSMUSG00000055301 |
| Xenobiotics biodegradation and metabolism | Metabolism of xenobiotics by cytochrome P450 | 1 | 83 | ko00980 | ENSMUSG00000055301 |
| Xenobiotics biodegradation and metabolism | Drug metabolism - other enzymes | 1 | 97 | ko00983 | ENSMUSG00000009350 |
