# Supplementary material for: Maternal obesity may disrupt offspring metabolism by inducing oocyte genome hyper-methylation via increased DNMTs
Source: eLife. 2024 Dec 6;13:RP97507. doi: 10.7554/eLife.97507 (PMC11623932; doi:10.7554/eLife.97507)
Supplement: Supplementary file 3. [file elife-97507-supp3.docx]

**Table S3 DMRs methylation status of metabolism-relative genes**

|  |  |  |
| --- | --- | --- |
| **Gene ID** | **Symbol** | **Hyper/Hypo** |
| ENSMUSG00000014245 | Pigl | Hyper |
| ENSMUSG00000062908 | Acadm | Hyper |
| ENSMUSG00000055301 | Adh7 | Hyper |
| ENSMUSG00000020346 | Mgat1 | Hyper |
| ENSMUSG00000021263 | Degs2 | Hyper |
| ENSMUSG00000021360 | Gcnt2 | Hyper |
| ENSMUSG00000021699 | Pde4d | Hyper |
| ENSMUSG00000022450 | Ndufa6 | Hyper |
| ENSMUSG00000022562 | Oplah | Hyper |
| ENSMUSG00000023913 | Pla2g7 | Hyper |
| ENSMUSG00000024039 | Cbs | Hyper |
| ENSMUSG00000024365 | Cyp21a1 | Hyper |
| ENSMUSG00000024799 | Tm7sf2 | Hyper |
| ENSMUSG00000025357 | Dgka | Hyper |
| ENSMUSG00000028894 | Inpp5b | Hyper |
| ENSMUSG00000029063 | Nadk | Hyper |
| ENSMUSG00000028463 | Car9 | Hyper |
| ENSMUSG00000028671 | Gale | Hyper |
| ENSMUSG00000028684 | Urod | Hyper |
| ENSMUSG00000002028 | Kmt2a | Hypo |
| ENSMUSG00000017713 | Tha1 | Hypo |
| ENSMUSG00000049721 | Gal3st1 | Hypo |
| ENSMUSG00000057342 | Sphk2 | Hypo |
| ENSMUSG00000061838 | Suclg2 | Hypo |
| ENSMUSG00000042638 | Gucy2c | Hypo |
| ENSMUSG00000043998 | Mgat2 | Hypo |
| ENSMUSG00000039936 | Pik3cd | Hypo |
| ENSMUSG00000021684 | Pde8b | Hypo |
| ENSMUSG00000021957 | Tkt | Hypo |
| ENSMUSG00000026807 | Ak8 | Hypo |
| ENSMUSG00000023805 | Synj2 | Hypo |
| ENSMUSG00000025477 | Inpp5a | Hypo |
| ENSMUSG00000025153 | Fasn | Hypo |
| ENSMUSG00000027695 | Pld1 | Hypo |
| ENSMUSG00000031387 | Renbp | Hypo |
