# Supplementary material for: Maternal obesity may disrupt offspring metabolism by inducing oocyte genome hyper-methylation via increased DNMTs
Source: eLife. 2024 Dec 6;13:RP97507. doi: 10.7554/eLife.97507 (PMC11623932; doi:10.7554/eLife.97507)
Supplement: Supplementary file 4. [file elife-97507-supp4.docx]

**Table S4 Binding sites of CREB1 on sequences of DNMTs**

| **Name** | **Relative score** | **Sequence ID** | **Start** | **End** | **Strand** | **Predicted sequence** |
| --- | --- | --- | --- | --- | --- | --- |
| Chr12: 3976409:3977697 |  |  |  |  |  |  |
| MA0018.2.CREB1 | 0.8299 | DNMT3a | 431 | 438 | - | TGAGGCCT |
| MA0018.2.CREB1 | 0.8092 | DNMT3a | 42 | 49 | + | TGAGGTCC |
| MA0018.2.CREB1 | 0.8012 | DNMT3a | 1255 | 1262 | - | CGAGGCCA |
| 12:3875426:3875897 |  |  |  |  |  |  |
| MA0018.2.CREB1 | 0.8299 | DNMT3a | 372 | 379 | - | TGAGGCCT |
| Chr12: 3977963:3978629 |  |  |  |  |  |  |
| MA0018.2.CREB1 | 0.8901 | DNMT3a | 129 | 136 | + | TGAGGCCA |
| Chr9: 20864302:20865039 |  |  |  |  |  |  |
| MA0018.2.CREB1 | 0.9342 | DNMT1 | 408 | 415 | + | TGAGGTCA |
| MA0018.2.CREB1 | 0.8642 | DNMT1 | 408 | 415 | - | TGACCTCA |
| Chr10: 77897008:77897613 |  |  |  |  |  |  |
| MA0018.2.CREB1 | 0.8574 | DNMT3l | 336 | 343 | + | TGAGGTGA |
| MA0018.2.CREB1 | 0.8092 | DNMT3l | 507 | 514 | - | TGAGGGCA |
| MA0018.2.CREB1 | 0.8012 | DNMT3l | 405 | 412 | - | GGAGGCCA |

Note: Sequences are obtained from Cut & Tag assay. Chr: chromosome.
