# Supplementary material for: Maternal obesity may disrupt offspring metabolism by inducing oocyte genome hyper-methylation via increased DNMTs
Source: eLife. 2024 Dec 6;13:RP97507. doi: 10.7554/eLife.97507 (PMC11623932; doi:10.7554/eLife.97507)
Supplement: Supplementary file 5. [file elife-97507-supp5.docx]

**Table S5 Annotation of peaks at relative gene regions**

|  |  |  |  |  |  |  |  |  |  |  |  |
| --- | --- | --- | --- | --- | --- | --- | --- | --- | --- | --- | --- |
| **Peak** | **Chr** | **Start** | **End** | **Length** | **-Log10**  **(p value)** | **Annotation** | **GeneChr** | **Gene**  **Start** | **Gene**  **End** | **Gene**  **Length** | **SYMBOL** |
| peak2_1593 | chr10 | 77897008 | 77897613 | 606 | 12.4365 | Intron (ENSMUST00000138785.8/54427, intron 13 of 13) | chr10 | 77891169 | 77899449 | 8281 | Dnmt3l |
| peak2_2951 | chr12 | 3976409 | 3977697 | 1289 | 10.9896 | Distal Intergenic | chr12 | 3857160 | 3963491 | 106332 | Dnmt3a |
| peak2_2952 | chr12 | 3977963 | 3978629 | 667 | 8.99516 | Distal Intergenic | chr12 | 3857160 | 3963491 | 106332 | Dnmt3a |
| peak2_415 | chr12 | 3875426 | 3875897 | 472 | 12.1207 | Intron (ENSMUST00000173700.2/13435, intron 1 of 1) | chr12 | 3857160 | 3963491 | 106332 | Dnmt3a |
| peak2_14239 | chr9 | 20864302 | 20865039 | 738 | 10.9407 | Promoter (<=1kb) | chr9 | 20818505 | 20864275 | 45771 | Dnmt1 |

Note: Chr, chromosome.
