# Supplementary material for: Maternal obesity may disrupt offspring metabolism by inducing oocyte genome hyper-methylation via increased DNMTs
Source: eLife. 2024 Dec 6;13:RP97507. doi: 10.7554/eLife.97507 (PMC11623932; doi:10.7554/eLife.97507)
Supplement: Supplementary file 6. [file elife-97507-supp6.docx]

**Table S6 Percentage of ingredient of diets.**

| Class description | D12492 (%) | Normal Diet (%) |
| --- | --- | --- |
| Protein | 26.23 | 24.02 |
| Carbohydrate | 25.56 | 29.75 |
| Fiber | 6.46 | ≤5 |
| Fat | 34.89 | 12.95 |
| Mineral | 6.46 | 4.42 |
| Vitamin | 0.39 | 0.36 |
| Dye | 0.0065 | 0 |
| H2O |  | ≤10 |

Note: for D12492, the percentage is calculated using ingredient weight/773.85 (g) ×100%; for normal diet, the percentage is calculated using ingredient weight/1000 (g) ×100%.
